# Organ-specific extracellular matrix directs trans-differentiation of mesenchymal stem cells and formation of salivary gland-like organoids in vivo

**DOI:** 10.1186/s13287-022-02993-y

**Published:** 2022-07-15

**Authors:** Olivia N. Tran, Hanzhou Wang, Shengxian Li, Andrey Malakhov, Yuyang Sun, Parveez A. Abdul Azees, Aaron O. Gonzalez, Brian Cao, Milos Marinkovic, Brij B. Singh, David D. Dean, Chih-Ko Yeh, Xiao-Dong Chen

**Affiliations:** 1 Department of Comprehensive Dentistry, University of Texas Health Science Center at San Antonio, San Antonio, TX 78229-3900 USA; 2 Department of Biomedical Engineering, University of Texas at San Antonio, San Antonio, TX 78249 USA; 3 Department of Endocrinology, Renji Hospital, School of Medicine, Shanghai Jiaotong University, Shanghai, 200126 People’s Republic of China; 4 Department of Periodontics, University of Texas Health Science Center at San Antonio, San Antonio, TX 78229-3900 USA; 5 Geriatric Research, Education and Clinical Center, South Texas Veterans Health Care System, San Antonio, TX 78229-4404 USA; 6 Present Address: Research Service, South Texas Veterans Health Care System, San Antonio, TX 78229-4404 USA

**Keywords:** Salivary gland, Regeneration, Mesenchymal stem cells, Stem cell niche, Cell trans-differentiation

## Abstract

**Background:**

Current treatments for salivary gland (SG) hypofunction are palliative and do not address the underlying cause or progression of the disease. SG-derived stem cells have the potential to treat SG hypofunction, but their isolation is challenging, especially when the tissue has been damaged by disease or irradiation for head and neck cancer. In the current study, we test the hypothesis that multipotent bone marrow-derived mesenchymal stem cells (BM-MSCs) in a rat model are capable of trans-differentiating to the SG epithelial cell lineage when induced by a native SG-specific extracellular matrix (SG-ECM) and thus may be a viable substitute for repairing damaged SGs.

**Methods:**

Rat BM-MSCs were treated with homogenates of decellularized rat SG-ECM for one hour in cell suspension and then cultured in tissue culture plates for 7 days in growth media. By day 7, the cultures contained cell aggregates and a cell monolayer. The cell aggregates were hand-selected under a dissecting microscope, transferred to a new tissue culture dish, and cultured for an additional 7 days in epithelial cell differentiation media. Cell aggregates and cells isolated from the monolayer were evaluated for expression of SG progenitor and epithelial cell specific markers, cell morphology and ultrastructure, and ability to form SG-like organoids in vivo.

**Results:**

The results showed that this approach was very effective and guided the trans-differentiation of a subpopulation of CD133-positive BM-MSCs to the SG epithelial cell lineage. These cells expressed amylase, tight junction proteins (Cldn 3 and 10), and markers for SG acinar (Aqp5 and Mist 1) and ductal (Krt 14) cells at both the transcript and protein levels, produced intracellular secretory granules which were morphologically identical to those found in submandibular gland, and formed SG-like organoids when implanted in the renal capsule in vivo.

**Conclusions:**

The results of this study suggest the feasibility of using autologous BM-MSCs as an abundant source of stem cells for treating SG hypofunction and restoring the production of saliva in these patients.

## Background

Salivary gland (SG) hypofunction, often a consequence of radiation therapy for head and neck cancer, Sjögren’s syndrome, aging or various systemic diseases, impacts millions of people in the USA every year [1–3]. SG secretions (i.e., saliva) contain multiple antimicrobial agents/factors, buffer systems, lubricants, and digestive enzymes which work together to maintain the dentition, oral tissues, and initiate digestion [3]. Thus, patients with SG hypofunction frequently suffer from severe oral diseases and/or compromised oral function, which lead to poor quality of life [3]. Current therapies for SG hypofunction are primarily palliative and do not address the underlying cause(s) or progression of the disease process [1]. Since adult SGs are highly differentiated tissues/glands, they have limited regenerative capacity once damaged. In an effort to restore SG function, regenerative medicine approaches are being actively pursued to manage and/or treat these patients [4].

A number of strategies for restoring SG function have been proposed, including: (1) transfection of therapeutic genes (e.g., aquaporin-1 gene) into residual salivary acinar or ductal cells [5, 6], (2) replacement of the entire SG with a functional artificial tissue [7–9], and (3) regeneration of SG tissue in situ [10–14]. The first approach via transient gene transfer has been shown to produce a temporary improvement in gland function in animal models and recent results in a human clinical trial have been encouraging. In contrast, results employing the other two approaches have been developing more slowly since they require complex strategies and an advanced understanding of stem cell biology and tissue engineering.

A large quantity of stem cells is required for SG regenerative medicine strategies. However, resident stem cells in SG tissue are not well defined, available in very limited numbers, and difficult to obtain/access, especially from patients receiving irradiation for head and neck cancer or afflicted with SG disorders [15–17]. Over the last decade, SG cells, expressing c-Kit, CD133, and Musashi1, have been shown to be capable of partially restoring radiation-damaged SG function [10, 11, 13]. Culture systems are also an important component, but it has been difficult to expand sufficient numbers of cells that retain their SG stem cell properties for basic research and therapeutic applications [18]. Here, we propose a new strategy for producing sufficient numbers of SG stem cells to repair or regenerate damaged SG by inducing the differentiation of bone marrow-derived mesenchymal stem cells (BM-MSCs) to the SG epithelial cell lineage by incubation with a SG-specific microenvironment (i.e., submandibular gland extracellular matrix [SMG-ECM]).

As the “gold standard,” BM-MSCs are relatively easy to obtain from patients and expand for autologous transplantation [19]. In addition, they are multipotent and can differentiate into a multitude of distinct cell types [20–22]. Although there is evidence that BM-MSCs are capable of trans-differentiation to the ectodermal or endodermal lineage [22], there is still conflicting evidence as to whether BM-MSCs are able to differentiate into functional SG epithelial cells using current published approaches [23]. In the present study, we tested the *hypothesis* that BM-MSCs, treated with tissue-specific ECM from decellularized SMG organ, were able to trans-differentiate to the SG epithelial cell lineage. This approach is based on our prior studies showing that the use of native tissue-specific ECMs directs multipotent stem cell differentiation to the same lineage as that of the ECM [24, 25].

## Methods

### Animals

*Male Lewis rats*, 3–5 months old, were purchased from Envigo (Indianapolis, IN, USA) and used as a source of SMGs and bone marrow. *Immunodeficient female mice*, NIH III HO, 3–4 months old, purchased from Charles River (Wilmington, NC, USA), were used for renal capsule implantation studies. Rats and mice were fed standard rodent chow and water ad libitum and housed in an AAALAC-accredited vivarium with regulated temperature (20–24 °C) and 12-h light/dark cycle. All use of the animals complied with the ARRIVE guidelines, and all procedures performed on the animals complied with PHS/NIH Animal Care and Use Guidelines and were approved by the IACUC at the University of Texas Health Science Center at San Antonio.

### Decellularization of SMGs and preparation of SMG-ECM

**Fig. 1:**
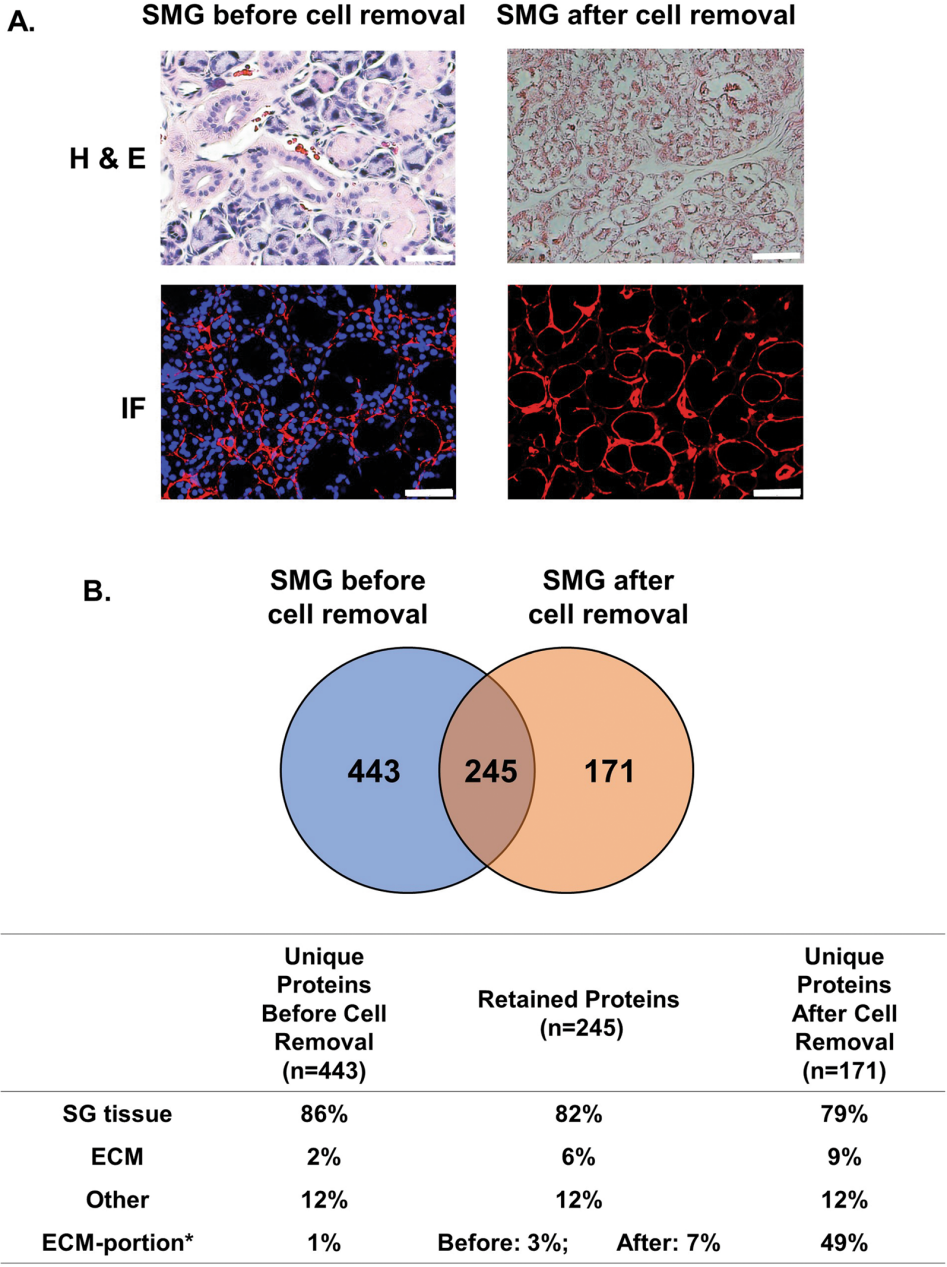

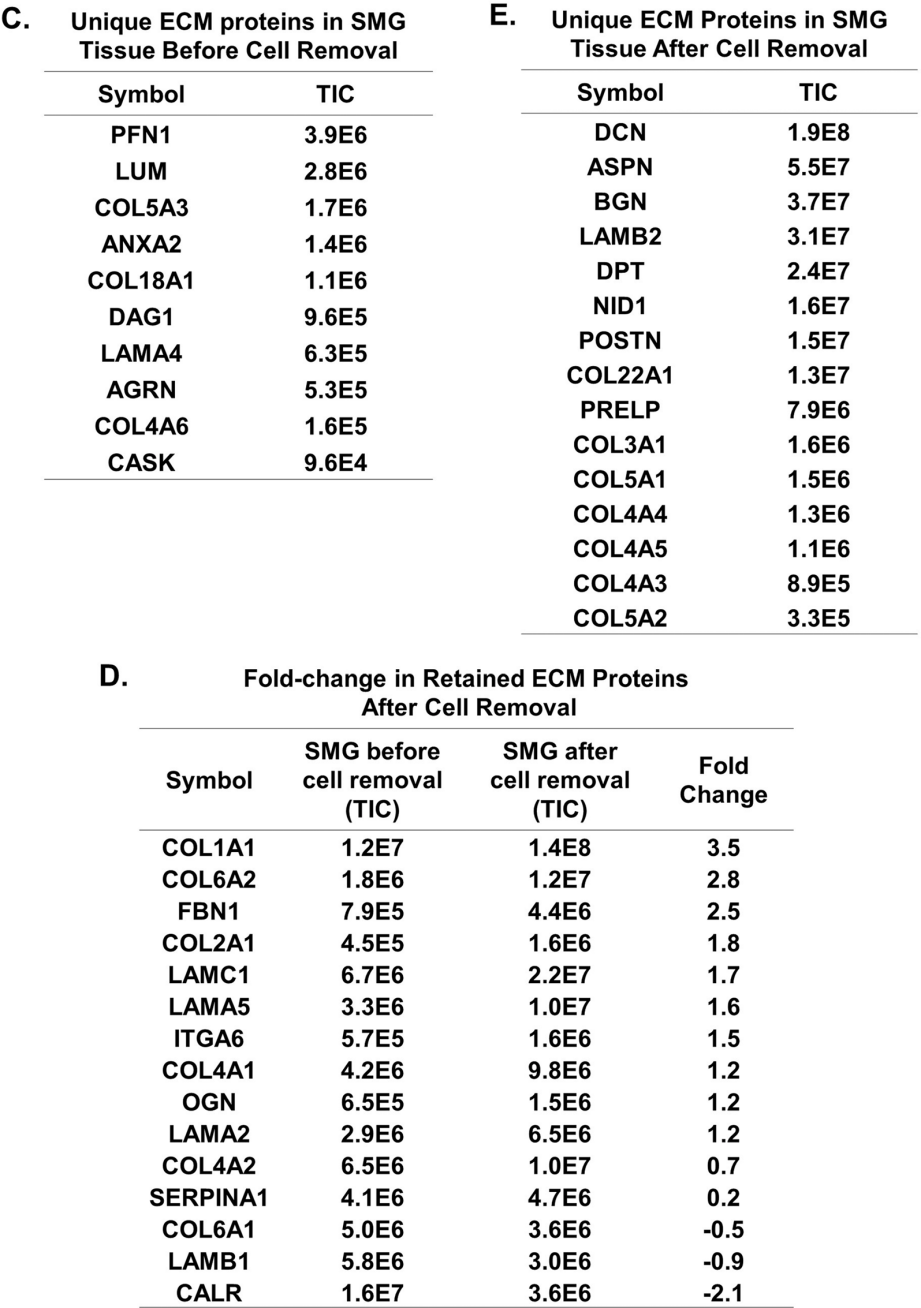
Light and immunofluorescence microscopy and proteomic analysis showed that decellularization removed cell nuclei from SMG tissue and left ECM components intact. **A** Hematoxylin and eosin (H&E) staining of SMG tissue sections showed that decellularization removed the vast majority of cell nuclei (blue). Immunofluorescence (IF) staining for type IV collagen (red) showed that decellularization left the ECM intact, while DAPI staining (blue) showed that cell nuclei were removed. Staining with nonspecific isotype antibodies was used as a negative control (not shown). Bar: 50 µm. **B** Analysis of the protein components present in SMG tissue before and after decellularization using mass spectrometry. The Venn diagram shows the number of unique proteins identified in SMG before and after decellularization. The area of intersection shows the number of identical proteins retained in both samples. In the table, proteins identified by proteomic analysis have been classified based on their functionality (“SG tissue,” “ECM,” or “Other”) using the Jensen TISSUES database. The percent of proteins in each class is shown. Note: ECM data were further analyzed, using normalized total ion current (TIC), to obtain an estimate of the *quantity* of proteins present and a percent calculated; this is shown as “ECM-portion*.” Decellularization enriched the absolute quantity of ECM proteins present. **C** List of unique ECM proteins in SMG tissue before cell removal. The data are expressed as normalized TIC. **D** List of retained ECM proteins in SMG tissue after cell removal and a calculation of the relative fold change (log_2_ [TIC after cell removal/TIC before cell removal]). **E** List of unique ECM proteins in SMG tissue after cell removal. The data are expressed as normalized TIC

SMGs were obtained after humane euthanasia (10 glands from 5 rats; total weight 2–2.5 g) and stored dry in a − 80 °C freezer. On the day of decellularization, SMGs were thawed and adherent tissue and fat removed, followed by cutting into ~ 3 mm^3^ cubes with scissors and placing into a 50 mL conical tube containing 30 ml of decellularization buffer (8 mM CHAPS, 1.0 M NaCl, 25 mM disodium EDTA, and EDTA-free Pierce™ protease inhibitor [Pierce Biotechnology, Rockford, IL, USA] in phosphate-buffered saline [PBS]) [26, 27]. Decellularization was conducted for 72 h at 37 °C with buffer changes every 24 h. After the last buffer change, SMG tissue was rinsed with 40 ml of PBS containing penicillin/streptomycin (2%) for 48 h at 4 °C with solution changes every 12 h. To ensure removal of DNA and RNA, the minced tissues were treated with 90 U/ml benzonase (Sigma-Aldrich, St. Louis, MO, USA) for 1 h at 37 °C, followed by rinsing and gentle shaking with PBS containing 10% FBS for 12 h at 37 °C. After this treatment, the tissue was washed a second time with 40 ml of PBS for 48 h at 4 °C with a solution change every 12 h. At this stage, the gland was fully decellularized (= SMG after cell removal) and compared to the intact SMG in various studies (i.e., histology and proteomic analysis) as described in Fig. 1.

To prepare homogenates of SMG-ECM for use in treating BM-MSCs, remnant SMG tissue after decellularization was further minced into smaller pieces (~ 0.2 mm) using 5½” straight surgical scissors at room temperature and placed into a small tube containing 4–5 mL PBS on ice. A PolyTron homogenizer (KINEMATICA, Switzerland) was then used to homogenize the tissue until all pieces of the minced tissue were no longer visible (Speed setting at 19, 5 s/run × 3, with a 60 s period on ice between runs to avoid overheating). After homogenization, the preparation was designated “SMG-ECM.” Total protein in the homogenate was measured using a Bio-Rad DC protein assay (Bio-Rad Laboratories, Hercules, CA, USA). SMG-ECM homogenate was aliquoted and stored at − 80 °C until needed in the experiments.

### Proteomic analysis of SMG tissue before and after decellularization

For proteomic analyses, SMG tissue and decellularized SMG tissue (≈100 mg) were homogenized in 1 mL extraction buffer (50 mM sodium acetate, pH 5.8, containing 4 M guanidine HCl, 65 mM dithiothreitol, 10 mM disodium EDTA, and mini-protease inhibitor cocktail [Roche Diagnostics, Germany]) and then vigorously shaken for 24 h at room temperature. After centrifugation, the supernatant containing extracted proteins was precipitated with 5-volumes ethanol (− 20 °C, 1 h), centrifuged, washed with ethanol, and stored at − 80 °C.

Dried pellets of the extracted SMG tissue and decellularized SMG tissue were reconstituted in Laemmli buffer containing 50 mM dithiothreitol and boiled at 100 °C for 5 min before loading onto a standard (one dimensional) SDS-PAGE gel followed by electrophoresis. Proteins were identified on the gels by Coomassie Blue staining and released from the gel by in situ trypsin digestion. Digests were analyzed using capillary HPLC-electrospray ionization tandem mass spectrometry as previously described [25]. Mascot software (Matrix Science, Inc., Boston, MA) was used to search the resulting spectra against the SwissProt database. Cross-correlation of the Mascot results with X! Tandem and determination of protein and peptide identity probabilities were performed in Scaffold (Proteome Software). Protein identifications were accepted according to the following criteria: minimum number of peptides, 2; peptide probability, ≥ 95%; 1.0% false discovery rate protein threshold. Individual proteins, identified in the analysis, were classified ontologically as belonging to either “SG tissue” or “ECM” by the Enrichr gene analysis tool (https://maayanlab.cloud/Enrichr/) using Jensen TISSUES, a tissue-specific gene expression database (https://tissues.jensenlab.org/). To normalize the range of amplitudes in the MS spectra, we report proteomic data in terms of normalized total ion current (TIC) [28]. This method normalizes the area under the curve for all spectra in order to transform them to a common intensity range suitable for comparison [29].

### Isolation of bone marrow-derived mesenchymal stem cells (BM-MSCs)

After humane euthanasia, Lewis rat femurs were removed, cleaned of all muscle and adherent tissue, and the epiphyses removed using a bone rongeur. Bone marrow (BM) was flushed from the bone shafts into a 50 mL conical tube using a 10 cc syringe fitted with an 18-gauge needle and filled with 10 mL ice cold HBSS containing 5% FBS. BM cells were collected by centrifugation at 450× *g* for 5 min at 4 °C and the pellet re-suspended in 45 ml of alpha-MEM containing 20% FBS and 1% penicillin/streptomycin (= growth media). The re-suspended cells were seeded (1–1.5 × 10^8^ cells/dish) into three 100 mm tissue culture dishes containing 15 ml of growth media. Half media changes were performed on day 3 and day 5. After 7 days, passage 1 (P1) BM cells were detached by treating with collagenase type II (400 units/ml) (Worthington, Lakewood, NJ, USA) for 5–10 min at room temperature. The harvested cells, mainly containing MSCs (> 90% CD90 shown in Fig. 6A), were filtered through a 70 µm cell strainer (Fisher Scientific, cat. # 22363548, Fair Lawn, NJ, USA) to create a single cell suspension for subsequent experiments.

### Treatment of BM-MSCs with SMG-ECM to induce trans-differentiation and formation of cell aggregates

BM-MSCs (P1) were incubated for 1 h in 0.5 ml of SMG-ECM homogenate (2 mg/mL; 1 ng protein/cell based on the Bio-Rad DC protein assay) with gentle shaking at 37 °C. After incubation, the cell/SMG-ECM suspension was transferred to 3–6-well tissue culture plates (Corning, Kennebunk, ME, USA) using a ratio of 6 × 10^3^ cells/cm^2^ and cultured for 7 days in growth media at 37 °C. On day 7, cell aggregates that had attached to the culture plates were collected using a 200 µl pipette and stereomicroscope (Olympus SZX), transferred to a non-tissue culture-treated 12-well plate (Corning, cat. # 351143), and cultured for an additional 7 days in DMEM / F12 (1:1) media (Gibco Life Technologies, Grand Island, NY, USA) containing 15% FBS and 20 mM hydrocortisone (Sigma-Aldrich) (= epithelial cell induction media). On day 14 of culture, cell aggregates were harvested as described above and then used in characterization studies.

### Assay of cell proliferation using bromodeoxyuridine (BrdU) incorporation

Cell proliferation was assayed using a BrdU colorimetric ELISA kit (Sigma-Aldrich, Indianapolis, IN). On day 4 and 7 of culture, cell aggregates in the SMG-ECM-treated cultures were separated from the monolayer cells and transferred to a 12-well plate, along with cells from untreated cultures, containing 1 ml of culture media. For assay, cell aggregates, monolayer cells, and untreated monolayer cells were pulse-labeled for 3 h with BrdU (100 µl/well) in 1 ml media/well. Negative control cultures were treated with Mitomycin C (Sigma-Aldrich, St. Louis, MO) (50ug/ml) for 1 h before addition of BrdU; other controls included cells not incubated with BrdU. After labeling, the BrdU containing media were removed, the cells isolated with collagenase, and then counted with a hemocytometer. Cells (i.e., from cell aggregates and SMG-ECM-treated monolayer and untreated cells) were plated onto a 96 well plate (10^4^ cells/well in triplicate) and dried using a hair dryer for 15 min. Cells were fixed with FixDenat (200 µl/well) for 30 min., the fixative removed, 100 µl of Anti-BrdU-POD (peroxidase conjugated secondary antibody binding to BrdU) added to each well, incubated for 90 min at 25 °C, and then removed. Each well was rinsed 3 times with PBS. Substrate solution was then added (100 µl/well) for color development. Absorbance was measured using a SpectraMax M2 microplate reader (Molecular Devices) at 370–490 nm after 0, 5,10, 15, and 20 min. The optimal time for development was found to be 15 min for all samples.

### Assay of changes in calcium flux

Untreated monolayer cells and SMG-ECM-treated cell aggregates and monolayer cells were incubated with 2 μM fura-2AM (Millipore, cat. # 344905, Billerica, MA) at 37 °C for 45 min and then washed 2 times with Ca^2+^-free SES buffer (i.e., Standard External Solution, containing 10 mM HEPES, 120 mM NaCl, 5.4 mM KCl, 1 mM MgCl_2_, and 10 mM glucose, pH 7.4). For assay, Fura-2 fluorescence intensity of the loaded control cells was monitored with a CCD camera-based imaging system (Hamamatsu Photonics, Japan) mounted on an Olympus XL70 inverted microscope equipped with an Olympus 40× (1.3 NA) objective. Fura-2 dual excitation and emission were accomplished using 340- and 380-nm excitation filters and a 510-nm emission filter. Imaging data acquisition was accomplished using MetaFluor software (Molecular Devices, San Jose, CA). Fluorescence traces show intracellular calcium [Ca2+]i values from an average of at least 50 or more cells and are representative of results obtained in at least 3 individual experiments.

### Real time PCR analysis

Gene expression studies in untreated controls and SMG-ECM-treated cell aggregates and monolayer cells were performed after 7 or 14 days in culture. Cell aggregates were collected manually (as described above), while monolayer cells were released by collagenase digestion. In all three types of cells, RNA was isolated using Trizol reagent (Life Technologies, Carlsbad, CA, USA). cDNA was reverse transcribed from the extracted RNA (1 µg) using a High-Capacity cDNA Reverse Transcription Kit (ThermoFisher, Foster City, CA, USA) and quantified using SYBR Green. Ct values were normalized to GAPDH (e.g., 2^[− (Ct^Muc10^ − Ct^GAPDH^)]). Rat primers, listed below, were purchased commercially from ThermoFisher.

**Table:** 

*Rattus norvegicus* GAPDH, Forward	AGTGCCAGCCTCGTCTCATA
*R. norvegicus* GAPDH, Reverse	GAAGGGGTCGTTGATGGCAA
*R. norvegicus* Oprpn (*Muc10*), Forward	ATCTCCCACCAAGGAGCAAC
*R. norvegicus* Oprpn (*Muc10*), Reverse	GTGGGTTTTGGCTGGAAGTGA
*R. norvegicus* Bhlha15 (*Mist1*), Forward	GTTCCAACCAGGGTGATCCTTT
*R. norvegicus* Bhlha15 (*Mist1*), Reverse	TTGAATAAACCCAGCCCCGT
*R. norvegicus* keratin 14 (*Krt14*), Forward	GCAGAACCTCAATGACCGCT
*R. norvegicus* keratin 14 (*Krt14*), Reverse	CCAGGATCTTGCTCTTCAGGT
*R. norvegicus* Claudin 3 (*Cldn3*), Forward	GAGTGCTTTTCCTGTTGGCG
*R. norvegicus* Claudin 3 (*Cldn3*), Reverse	CCAGTTCCCATCTCTCGCTT
*R. norvegicus* Claudin 10 (*Cldn10*), Forward	CTTCCACACTACCCACCGAC
*R. norvegicus* Claudin 10 (*Cldn10*), Reverse	ATGTAACCATCCAGCGCCAG

### Analysis of cell surface marker expression using flow cytometry

Before analysis, cells were dissociated into single cells by treatment with collagenase type II (Worthington, Lakewood, NJ, USA) for 10 min at 37 °C. Cells were incubated with primary antibodies at room temperature for one hour or overnight at 4 °C and then washed twice with FACS buffer (HBSS + 5% FBS + 0.1% sodium azide) before incubation with secondary antibody for 1 h at 4 °C. Cells were subsequently washed two times with FACS buffer and then immediately analyzed using a BD Bioscience LSRII flow cytometer running FACSDiva software. Data were analyzed and figures created using FlowJo software. At least 10,000 events were measured in each sample and the percent positive cells (relative to isotype control) determined. The primary and secondary antibodies used were mouse anti-rat unconjugated CD90 (BD Biosciences, cat. # 554895, San Jose, CA, USA), mouse anti-rat unconjugated CD105 (ThermoFisher, cat. # MEM-226, Rockford, IL, USA), rabbit anti-rat CD133 unconjugated (ThermoFisher, cat. # PA5-38014, Rockford, IL, USA), goat anti-rabbit IgG H&I Alexa Fluor 647 (Abcam, cat. # ab150079, Cambridge, MA, USA), goat anti-mouse IgG (H + L) highly cross-adsorbed secondary antibody Alexa Fluor 647 (ThermoFisher, cat. # A-21236, Eugene, OR, USA).

### Light microscopic analysis of SMG and SMG-ECM-treated BM-MSCs

For microscopic analysis, untreated control cells and SMG-ECM-treated cell aggregates and monolayer cells were collected by centrifugation at 450× *g* for 5 min at 4 °C. Cell pellets were fixed using 4% paraformaldehyde, embedded in paraffin, sectioned to a thickness of 5 µm, and then stained with either hematoxylin and eosin (H&E) or periodic acid Schiff (PAS). For immunocytochemistry, paraffin sections were heated to 65 °C for 1 h, deparaffinized, and then rehydrated using routine histological methods. Antigen retrieval was performed by submerging the slides in hot (95 °C) 10 mM sodium citrate buffer (pH 6.0) and microwaving at 20% power for 3 min. After cooling to room temperature for 30 min, nonspecific binding was blocked by incubation in 10% donkey serum (Santa Cruz, Santa Cruz, CA) diluted in 0.3% Triton X-100 + PBS for 1 h; subsequently, primary antibody (1:50) diluted in 1% BSA-PBS containing 0.3% Triton X-100 was added to the slide and allowed to bind overnight at 4 °C. On the next day, slides were washed 3 times with PBS and then treated with fluorescently labeled conjugated secondary antibody (1:500), diluted in 1% BSA-PBS containing 0.3%Triton X-100, for 1 h at 4 °C. Slides were then washed three times with PBS, mounted in DAPI-containing media (Fluoroshield with DAPI, Sigma-Aldrich), and the sections overlaid with a glass coverslip. Paraffin-embedded rat SMG tissue sections were used as positive controls to confirm positive staining in the experimental samples. In addition, staining with nonspecific isotype antibodies was used as a negative control.

The primary and secondary antibodies used in these studies were cross-reactive with rat and included the following: mouse Cytokeratin 14 monoclonal IgG3 antibody (Invitrogen, cat. # LL002), rabbit Claudin 3 polyclonal antibody (Invitrogen, cat. # 34-1700, Rockford, IL), rabbit Aquaporin 5 polyclonal antibody (Invitrogen, cat. # PA5-99403, Rockford, IL), mouse MIST1 monoclonal IgG1 antibody (Santa Cruz Biotechnology, cat. # sc-80984, Santa Cruz, CA), rabbit Claudin 10 polyclonal antibody (ThermoFisher, cat. # 38-8400, Rockford, IL), rabbit Amylase polyclonal antibody (Millipore Sigma, cat. # A8273, Saint Louis, MO), rabbit IgG isotype (Invitrogen, cat. # RI238244, Rockford, IL), mouse IgG1 isotype (BD Biosciences, cat. # 550878, San Jose, CA), mouse IgG3 isotype (BD Biosciences, cat. # 55034, San Jose, CA), goat anti-rabbit IgG (H&L) Alexa Fluor 647 (Abcam, cat. # ab150079, Cambridge, MA), and goat anti-mouse IgG Alexa Fluor 647 (ThermoFisher, cat. # A-21236, Eugene, OR).

### Renal capsule transplantation

BM-MSCs were incubated for 1 h with SG-ECM and then cultured for 14 days as described above to prepare cell aggregates. On the day of transplantation, cell aggregates (300–500 aggregates/transplantation site) were manually collected, transferred to PE50 tubing (Braintree Scientific Inc, Braintree, MA, USA), and maintained in a 50 ml conical tube on ice until transplantation. BM-MSCs treated with Matrigel (Corning), instead of SMG-ECM, were used as a control group.

Mice were anesthetized by isoflurane (1–5%) inhalation, which was maintained throughout the surgical procedure by verification via negative toe pinch. For access to the kidney, the animal was placed in ventral recumbence on a warmed operating surface and the surgical area prepared by alternating scrubs of betadine or chlorhexidine and isopropyl alcohol. A ~ 2 cm full thickness, longitudinal skin incision was created on the dorsal midline. Blunt dissection was used to locate the right or left kidney through the muscular body wall. The body wall tissue over the kidney was incised parallel to the long axis of the organ and exteriorized by applying pressure on either side using the thumb and forefinger. From this point forward, the exteriorized kidney was maintained on a sterile gauze pad and kept moist with PBS. A 0.25-cm incision was created in the kidney capsule and a sterile glass rod used to create a subcapsular space via blunt dissection. One end of the PE50 tubing, containing the transplants, was inserted into the space. A Hamilton syringe, fitted with a 26G needle connected to the tubing, was used to deliver the cell aggregates into the subcapsular space. After injection, the PE50 tubing was removed, leaving the implanted cells in place without any need to close the capsular incision. The kidney was then repositioned in the retroperitoneal space within the body. The peritoneum and body wall were closed with resorbable sutures and the skin closed with suture, wound clips, or staples.

After 14 and 30 days of implantation, the left kidney was collected and frozen in OCT or fixed overnight in 10% formalin for light microscopy (paraffin sections) or in phosphate-buffered 4% formaldehyde containing 1% glutaraldehyde for TEM. After fixation, the harvested tissues/implants were embedded and sectioned (paraffin or frozen) for staining with H&E, PAS, Alcian blue, and Trichrome (Pathology Services, UTHSCSA). In addition, other sections were prepared for immunohistochemical staining for Amylase, Cytokeratin 14, Cytokeratin 7, Claudin 3, Claudin 10, Mist1, and Aqp5. TEM Images were prepared by the Electron Microscopy Lab at UTHSCSA and viewed using a JEOL 1400 (JEOL, USA).

### Statistical analysis of the data

Results were expressed as the mean ± SD. Statistical significance between mean values was determined by one-way ANOVA followed by t test with statistical significance determined at *p* < 0.05. One-way ANOVA Power analysis was used to determine sample size (*a* = 0.05, (1–*β*) > 80%). All experiments contained 3–6 replicates/treatment group and were repeated 2–6 times using cells from different donors. Information for a specific experiment is described in the figure legends.

## Results

### Major structural components of the SMG-ECM were maintained after decellularization.

The decellularization protocol used in this study successfully removed cells from the rat SMG tissue and left the remaining ECM visually intact (Fig. 1A). Proteomic analysis of SMG tissue before and after decellularization was performed using mass spectrometry (MS) and individual protein components annotated ontologically as belonging to either “SG tissue” or “ECM” by the Enrichr gene analysis tool using Jensen TISSUES. Proteins not associated with “SG tissue” or “ECM” were classified as “Other.” Overall, decellularization removed about 64% of the proteins in SG tissue (443 out of 688) (Fig. 1B); of these, 86% were ontologically SG proteins, while only 2% belonged to the ECM (see Fig. 1C) and 12% to “Other.” Although 82% of the retained proteins and 79% of the unique proteins after decellularization were ontologically “SG tissue”-related, the absolute number of proteins was substantially reduced by the decellularization procedure. In contrast, almost all of the ECM proteins were either enriched or identified after cell removal (see Fig. 1D, E for fold change or relative quantity). Among the most enriched ECM proteins were structural and basement membrane proteins (e.g., types I, II, and VI collagens; laminins and fibrillin-1). There were 171 unique proteins identified in decellularized SMG (Fig. 1B), and 9% of these were ECM proteins (e.g., decorin and biglycan), which belong to the small leucine-rich proteoglycan (SLRPs) family (Fig. 1E). A quantitative assessment of ECM proteins was performed, shown as “ECM-portion,” which indicated that ECM proteins were enriched from 1 to 3% before to as much as 49% after decellularization (Fig. 1B).

### BM-MSCs incubated with SMG-ECM formed cell aggregates

**Fig. 2:**
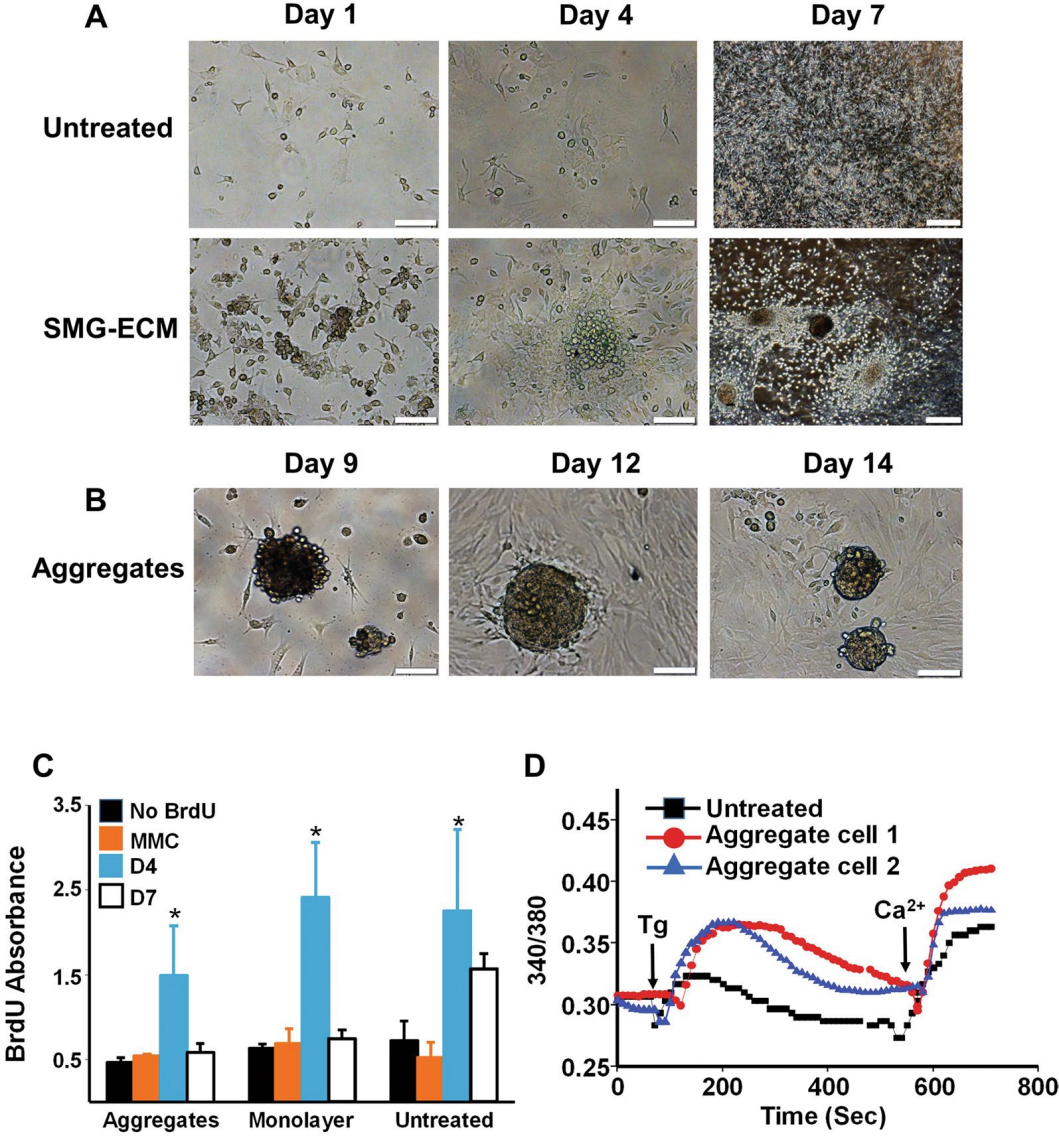
BM-MSCs incubated with SMG-ECM formed cell aggregates during culture for 7–14 days. **A** BM-MSCs were either untreated or treated with SMG-ECM for 1 h at 37 °C before transfer to a standard tissue culture dish and culture for up to 7 days. Over time, the untreated cells formed a monolayer across the surface of the dish, while the treated cells formed cell aggregates as well as a monolayer. Bar: 100 µm (2 left panels), 200 µm (far right panels). **B** After culture for 7 days, as described in **A**, cell aggregates were hand-picked under a dissecting microscope, transferred to a fresh tissue culture dish, and the cultures continued for an additional 7 days in epithelial cell differentiation media (i.e., 14 days total). During this second 7-day culture, cell aggregate organization was maintained. Bar: 100 µm. **C** BrdU assay was used to measure the proliferation of BM-MSCs that were untreated (Untreated) or treated with SMG-ECM and formed aggregates (aggregates) or a monolayer (monolayer) of cells during culture for 4 or 7 days. Negative control cultures either did not receive BrdU (No BrdU) or were treated with mitomycin C (MMC) to block proliferation. Data are the mean ± SD from a representative experiment. *p < 0.05 versus no BrdU and MMC groups; each experimental group contained an *n* = 4–6 and the experiment was repeated 3 times with MSCs from different donors. **D** Comparison of calcium mobilization in untreated and SMG-ECM-treated cell aggregates (50–100 cells) in the presence of thapsigargin (Tg in calcium-free media) followed by addition of external calcium (1 mM). The results were obtained using a minimum of 50 cells and are representative of those obtained in 3 individual experiments using cells from different donors

When BM-MSCs were pretreated with decellularized SMG-ECM in cell suspension for one hour and then transferred to a tissue culture plate and cultured for 7 days, the resulting cell cultures contained both cell aggregates and a cell monolayer (Fig. 2A). In contrast, untreated BM-MSCs only formed a cell monolayer (Fig. 2A). On day 7, cell aggregates were hand-selected under a dissecting microscope, transferred to a new culture dish, and the cultures continued in epithelial cell differentiation media for an additional 7 days (Fig. 2B). At the end of culture on day 14, there were approximately 2–4 aggregates/cm^2^ with an average perimeter of 326.5 ± 137.4 µm and containing 402 ± 135 cells/aggregate.

To determine if cell aggregate formation involved cell proliferation, we performed a pulse-label BrdU assay on days 4 and 7 of culture (Fig. 2C). Aggregates collected on day 4 incorporated more BrdU than those on day 7, indicating a peak in cell proliferation on that day (Fig. 2C). Untreated cells on TCP and monolayer cells treated with SMG-ECM displayed similar growth kinetics and BrdU incorporation patterns on day 4 of culture as the aggregates.

We next evaluated if cell aggregates were able to maintain SG cell physiology by assessing calcium mobilization using thapsigargin-induced calcium release. Thapsigargin is an inhibitor of endoplasmic reticulum (ER) Ca^2+^-ATPase and is used to empty the intracellular ER Ca^2+^ pool. Compared to untreated cells, cells in aggregates had a higher level of intracellular calcium release followed by an increase in calcium influx (Fig. 2D), indicating that aggregate physiology is distinct from that of the untreated cells and this increase in calcium could assist in salivary gland function as previously shown [30].

### Cell aggregates expressed SG progenitor cell-related transcripts

**Fig. 3:**
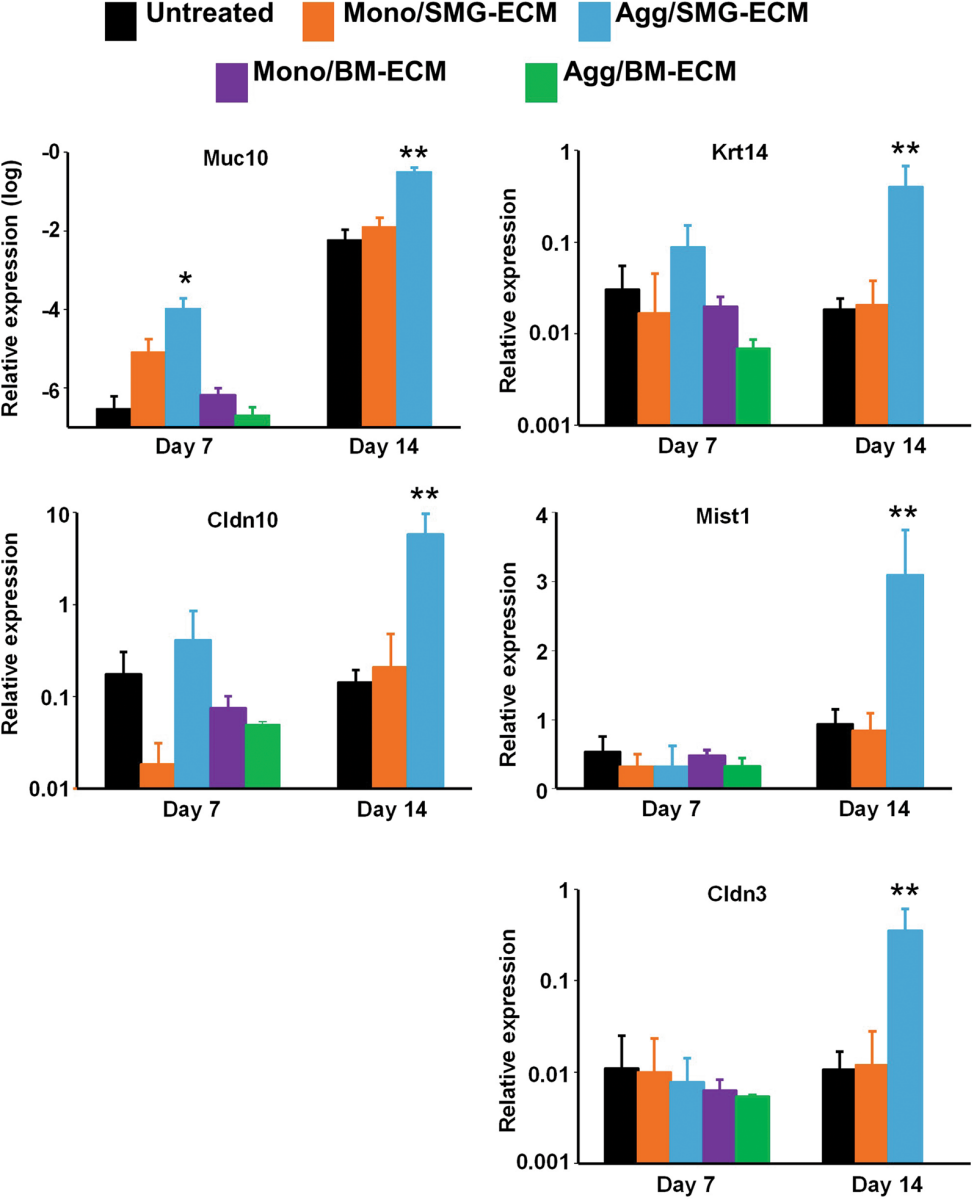
BM-MSCs treated with SMG-ECM formed cell aggregates expressing SG epithelial cell-related transcripts. BM-MSCs were either untreated (untreated) or treated with SMG-ECM or BM-ECM, as described in Fig. 2, for 7 or 14 days. RT-PCR was used to measure the expression of SG epithelial cell lineage markers in untreated cells or cells treated with SMG-ECM or BM-ECM and formed aggregates (Agg/SMG-ECM or Agg/BM-ECM) or a monolayer (Mono/SMG-ECM or Mono/BM-ECM). Data are the mean ± SD from a representative experiment. *p < 0.05, versus all other treatment groups on day 7; **p < 0.001, versus all other treatment groups on day 14. Each experiment contained 3 replicates, and each experiment was repeated 4–6 times with cells from different donors

Expression of SG progenitor and epithelial cell biomarkers [i.e., Mucin 10 (*Muc10)*, Cytokeratin 14 (*Krt14*), Mist1 (*Mist1*), and Claudin 3 (*Cldn3*) and Claudin 10 (*Cldn10*)] was used to assess the differentiation state of cells that had been untreated or treated with SMG-ECM or BM-ECM (Fig. 3). After 7 days in culture, *Muc10* was the only gene that was significantly increased in cell aggregates. Further, there were no significant changes in expression for any of the genes assayed in the SMG-ECM treated or untreated *monolayer* cells. To assess the specificity of the BM-MSC response to SMG-ECM, we treated BM-MSCs with bone marrow-derived ECM (BM-ECM) which has been shown to promote retention of stem cell properties (i.e., “stemness”) [31–33]. Although BM-ECM-treated BM-MSCs also formed aggregates, they did not express *Muc10*. By day 14, SMG-ECM-treated cell aggregates not only expressed a high level of *Muc10*, but all of the other SG epithelial cell lineage biomarkers. In contrast, SMG-ECM treated or untreated *monolayer* cells did not display enhanced expression for any of the SG-lineage biomarkers.

### Cell aggregate morphology and ultrastructure displayed SMG tissue-like characteristics

**Fig. 4:**
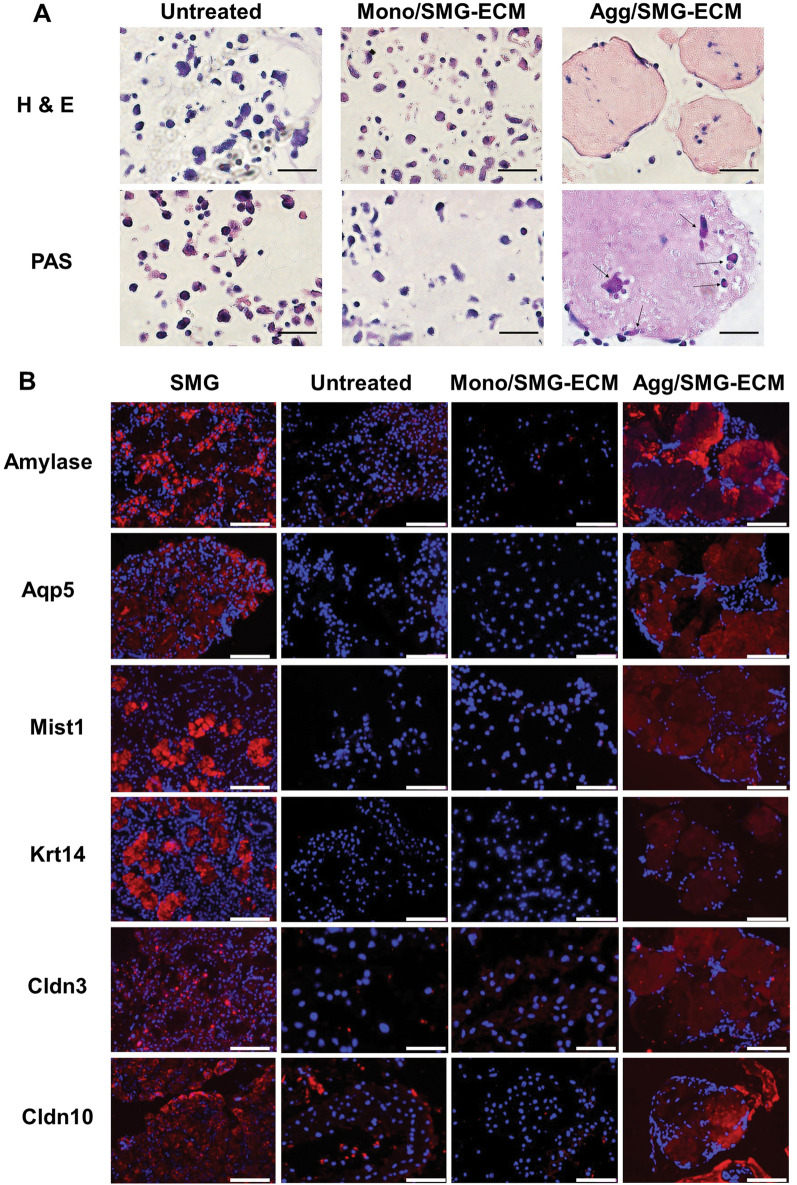
Histological analysis of SMG-ECM-treated cultures reveals that aggregates display an expression pattern different from monolayer cells. **A** Paraffin sections of cells from 14 day cultures (i.e., untreated BM-MSCs [Untreated], SMG-ECM-treated aggregates [Agg/SMG-ECM], and monolayer [Mono/SMG-ECM] cells) were prepared and stained with H&E or PAS. PAS-positive cells are identified with arrows in the figure. Bar: 50 µm. **B** Immunofluorescence staining of cells prepared as in **A** and rat SMG tissue (positive control) was used to evaluate the presence and localization of amylase, Aqp5, Mist1, Krt14, Cldn3, and Cldn10 protein. Staining with nonspecific isotype antibody was used as a negative control (not shown). Bar: 50 µm

Aggregates from 14-day cultures, stained with H&E, revealed the presence of cells inside and on the surface, while PAS-positive staining suggested that glycoproteins or mucin-like substances were present in the cells (Fig. 4A). Immunohistochemistry also detected the presence of amylase, aquaporin 5-positive (Aqp5^+^) and Mist 1^+^acinar-like cells, Krt14^+^ duct-like cells, and Cldn3 and Cldn10 tight junction proteins in SMG-ECM-treated aggregates (Fig. 4B). Morphologically, cell nuclei were primarily found at the periphery, as is typical of secretory units. However, none of these histologic or morphologic markers was detected in untreated cells, SMG-ECM-treated monolayer cells, or cell aggregates collected after 7 days in culture. Rat SMG tissue was used as a positive control, confirming that we were able to specifically identify salivary secretory units, ducts, and tight junction proteins in these cultures (Fig. 4B).

**Fig. 5:**
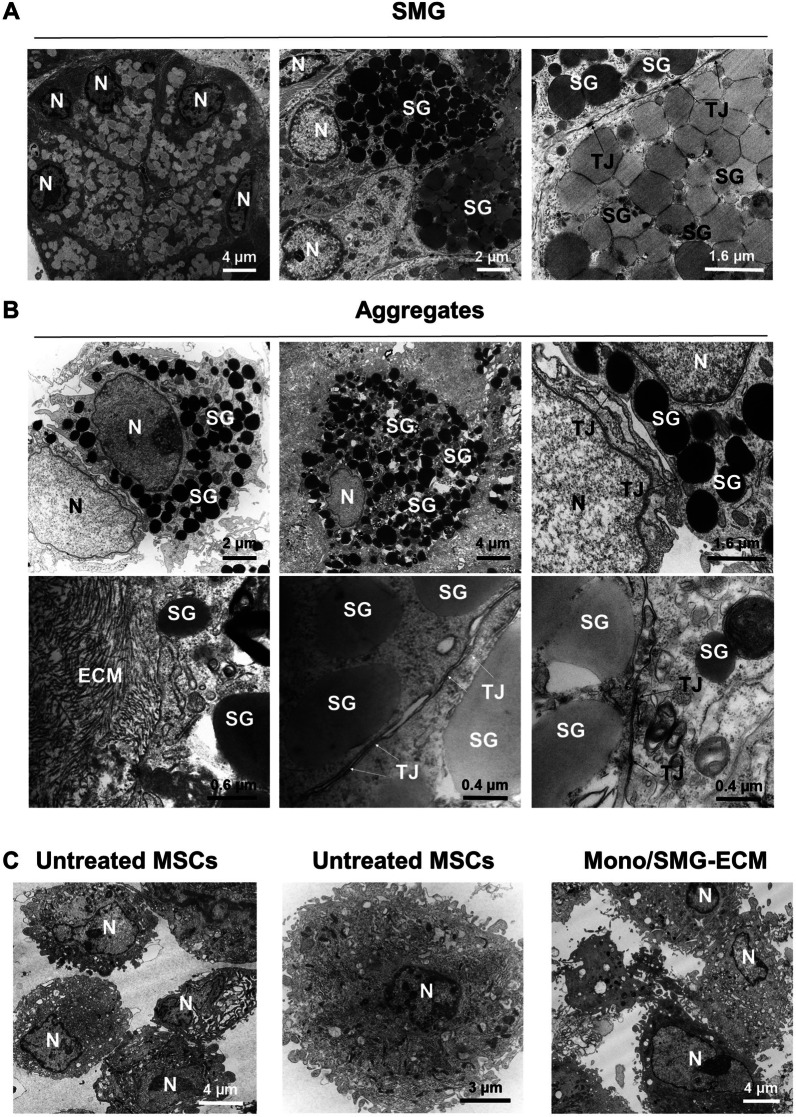
Ultrastructural characteristics of rat SMG tissue and SMG-ECM-treated aggregates were remarkably similar. **A** Transmission electron micrographs of rat SMG (positive control tissue) thin sections. N: cell nucleus; SG: secretory granule (note: there are two types: electron dense and less electron dense); TJ: tight junction. **B** Transmission electron micrographs of SMG-ECM-treated cell aggregates formed during 14 days in culture. Note the presence of structures (e.g., electron dense and less electron dense secretory granules; formation of tight junctions; and location of nucleus near the cell membrane) found in the rat SMG tissue that can also be seen in the cell aggregates. **C** Transmission electron micrographs of monolayer (Mono/SMG-ECM) and untreated BM-MSCs. Note: scale bar distances are shown in each panel

The ultrastructure of cell aggregates was examined by TEM. Cells in SMG-ECM-treated aggregates had an ultrastructure that was very similar to that of acinar cells in rat SMG tissue (Fig. 5A) and remarkably contained secretory granule-like structures in the cytoplasm, nuclei located near the periphery against the cell membrane, and numerous tight junctions between adjacent cells (Fig. 5B). In addition, two populations of secretory granules (low and high electron density) were identified in cell aggregates that were similar to those in SMG tissue. The presence of these two types of secretory granules suggests that two differentiation states of acinar cell development (i.e., serous and mucous glands) are being recapitulated in vitro. Further, some cells can be observed to interact with collagen fibers in the ECM (Fig. 5B). It should be noted that no granule-like structures were found in the SMG-ECM-treated monolayer cells or untreated control cells (Fig. 5C).

### A subpopulation of CD133-positive BM-MSCs was responsible for cell aggregate formation in the presence of SMG-ECM

**Fig. 6:**
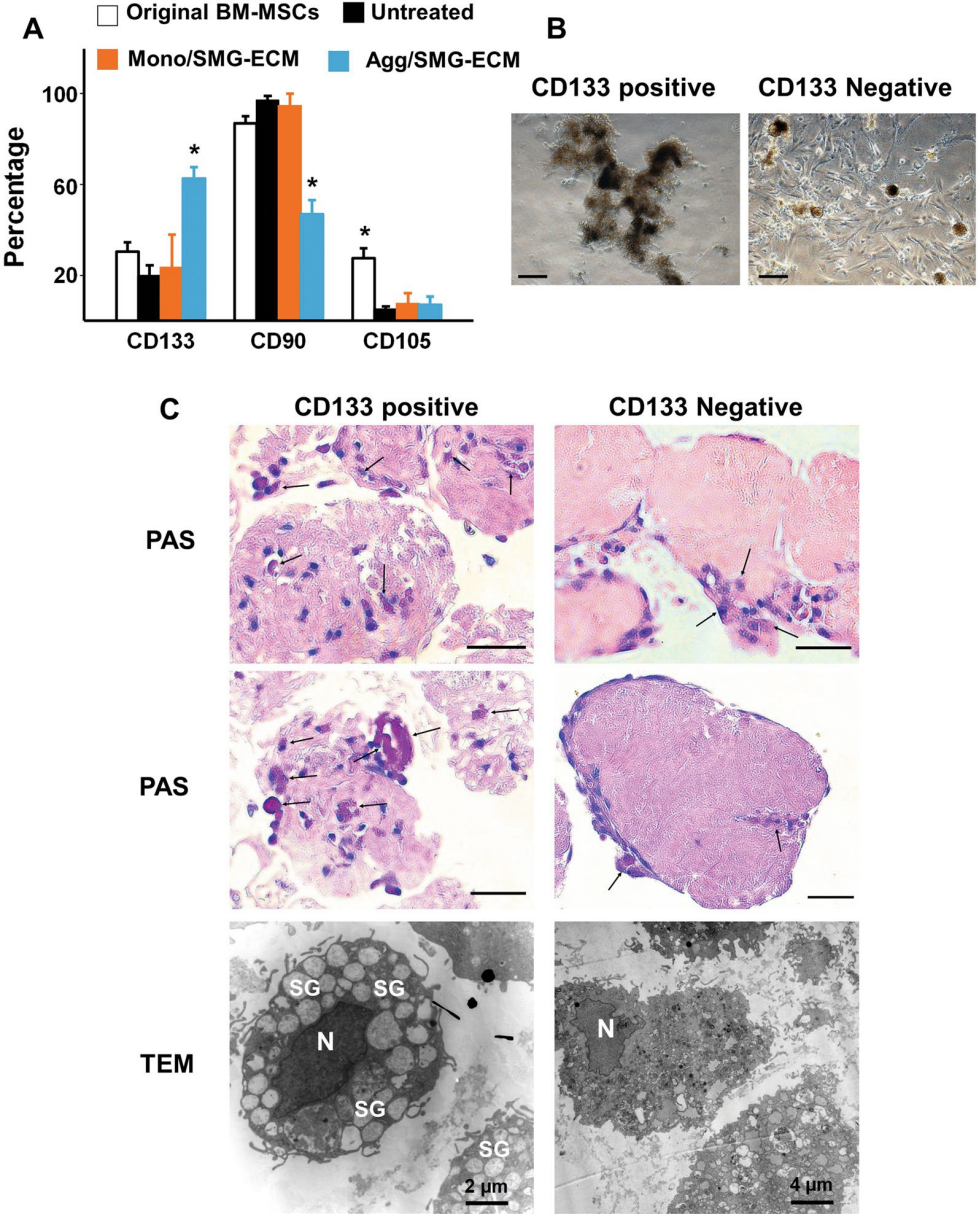
A subpopulation of BM-MSCs, expressing CD133, was associated with the formation of aggregates. **A** CD133, CD90, and CD105 expression was determined by flow cytometry in the original BM-MSCs (P1) (original), untreated BM-MSCs (untreated), and SMG-ECM-treated aggregates (Agg/SMG-ECM) or monolayer cells (Mono/SMG-ECM). Data are the mean ± SD from a representative experiment. The experiment was repeated 4 times, and in each experiment, 4 replicates (each using cells from a different donor) were used. *p < 0.05, versus all the other treatment groups for a specific marker. **B** Comparison of the aggregates formed by the CD133+ versus CD133− BM-MSC subpopulations after 14 days in culture. Bar: 200 µm. **C** Morphological comparison of the aggregates formed by the CD133  versus CD133− BM-MSC subpopulations. Arrow indicates PAS-positive cells, Bar: 50 µm; TEM shows differences in ultrastructure of the aggregates formed by the two BM-MSC subpopulations. (N: cell nucleus; SG: secretory granule), note: scale bar distances are shown in each panel

To determine whether a specific subpopulation of BM-MSCs was involved in cell aggregate formation, cell surface markers for MSCs, vascular cells, and epithelial cells (i.e., CD90, CD105, and CD133, respectively) were analyzed using flow cytometry. Cell aggregates and SMG-ECM treated and untreated monolayer cells were evaluated after 14 days in culture. The freshly prepared BM-MSC (P1) population was ~ 30% CD133+, ~ 87% CD90+, and ~ 28% CD105+. After 14 days in culture, the percent of CD133+ cells in aggregates increased to ~ 63%, while the percentage of CD90^+^ cells dropped to ~ 47%. In contrast, no significant change in the relative proportion of CD133+ and CD90+ cells in the untreated or treated monolayer cells and the P1 BM-MSCs was observed after 14 days (Fig. 6A). Interestingly, the percent of CD105+ cells was significantly decreased in the untreated and SMG-ECM-treated monolayer cells and cell aggregates as compared to P1 BM-MSCs.

The role of CD133+ cells in aggregate formation and trans-differentiation to the SG epithelial cell lineage was further explored by isolating a subpopulation of CD133+ cells from the P1 BM-MSC population using flow cytometry. For these studies, purified CD133+ or CD133− cells were treated with SMG-ECM, transferred to a fresh plate, and then cultured for 14 days as performed with the unfractionated MSCs. After 14 days in culture, the CD133+ subpopulation formed more aggregates that were larger than the CD133− subpopulation (Fig. 6B). PAS staining suggested that a greater number of PAS-positive cells, actively synthesizing glycoproteins or mucin-like substances, were found inside the ECM in cultures of CD133+ cells as compared to the CD133− cells (Fig. 6C). Further, TEM studies identified many cells within the aggregates formed in the CD133+ cultures containing intracellular secretory granule-like structures, while the same analysis of the CD133− cultures failed to identify any of these intracellular organelles (Fig. 6C).

### Cell aggregates formed SG-like organoids when implanted in a subrenal capsule model in vivo

**Fig. 7:**
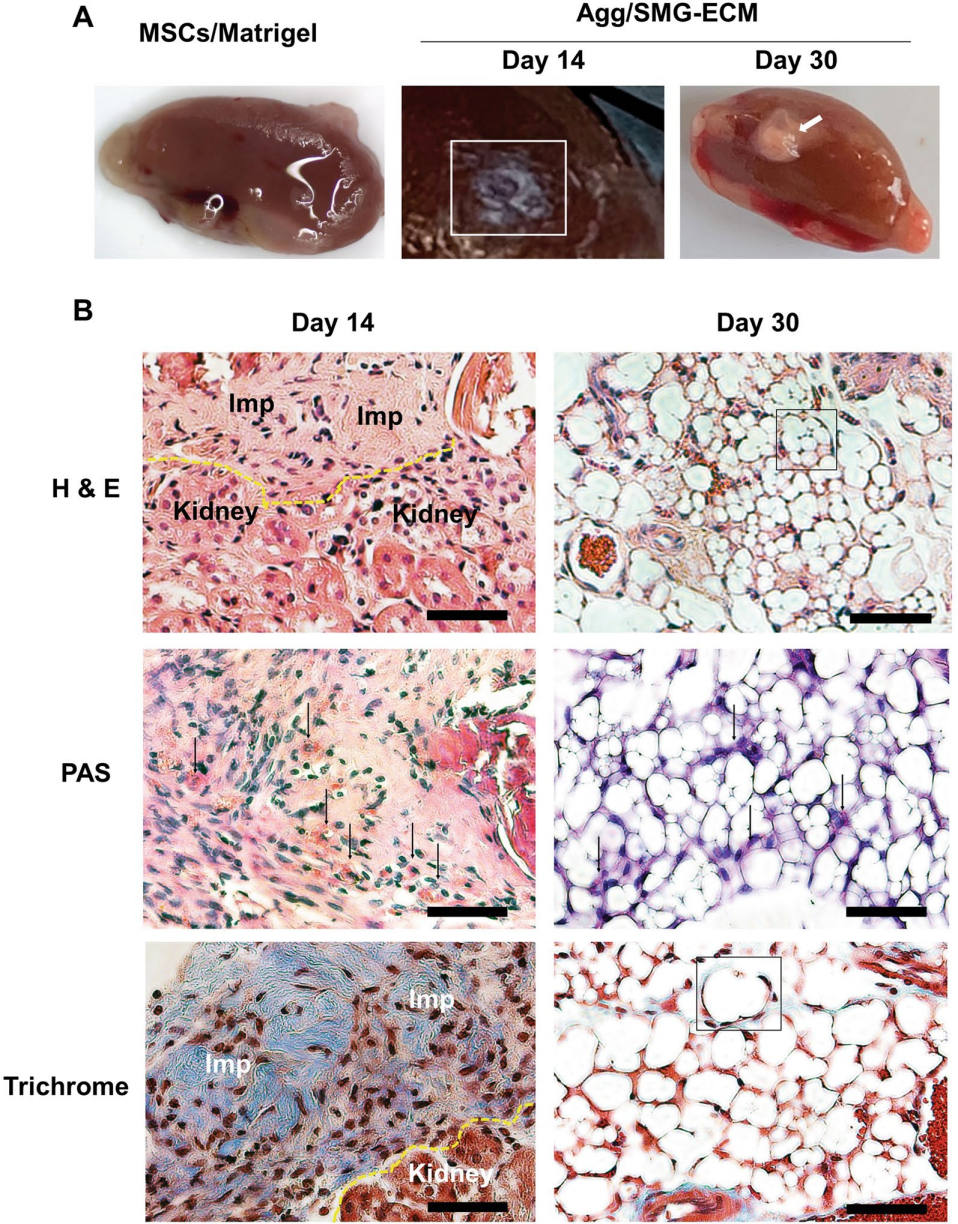

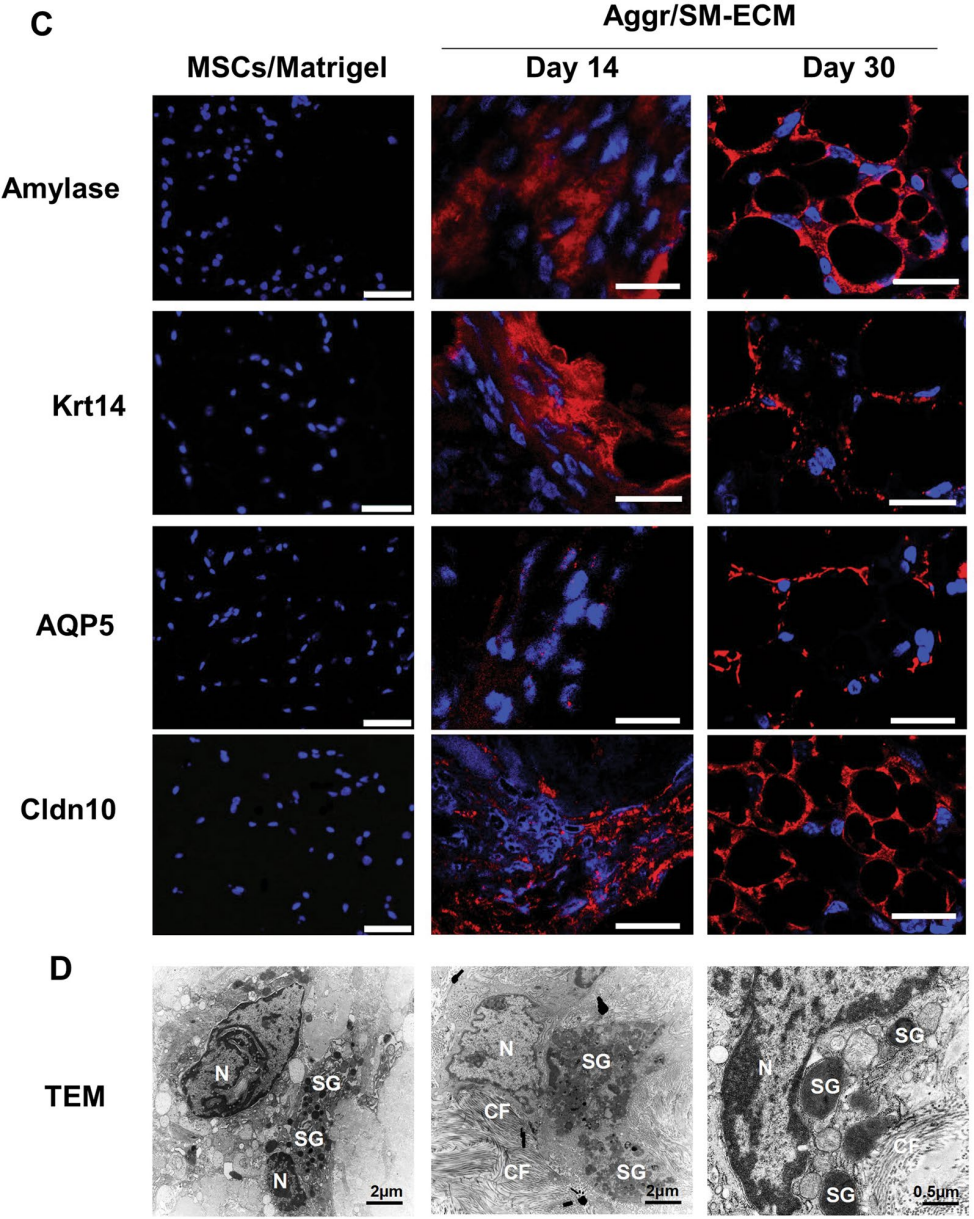
Implantation of aggregates in a subrenal capsule assay formed SG-like organoid structures in vivo. SMG-ECM-treated aggregates or BM-MSCs mixed with Matrigel (MSCs/Matrigel) were implanted under the kidney capsule in immunocompromised mice to study organoid development. The in vivo experiment was repeated 2 times, and there were 4 replicates of each treatment. Kidneys, containing the implants, were harvested after 14 and 30 days for histological analysis. **A** The shadow of a 14-day implant (i.e., transplantation of SMG-ECM-treated aggregates) is outlined by a white square in the figure, while a 30-day implant is indicated by a white arrow. BM-MSCs mixed with Matrigel (negative control) did not form any visible structures following implantation for 30 days. **B** Paraffin sections of the implants were stained with H&E, PAS, and Trichrome. In the H&E and Trichrome images of day 14 specimens, a dotted yellow line identifies the junction of the implant (Imp) with kidney tissue; in day 30 specimens, a black square outlines representative secretory acini-like units. In the PAS images, arrows identify positive-staining cells. Bar: 50 µm. **C** Immunofluorescence staining of specimens harvested from day 14 and 30 implants identified the presence of amylase, Krt14, Aqp5, and Cldn10. BM-MSCs mixed with Matrigel (MSCs/Matrigel) were implanted for 30 days as controls and processed in an identical fashion. Staining with nonspecific isotype antibody was used as a negative control (not shown). Bar: 50 µm (images of MSC/Matrigel implants); 20 µm (images of day 14 and 30 Agg/SM-ECM implants). **D** TEM images of the 30 day implants (Agg/SMG-ECM) reveal the ultrastructural organization of the forming SG organoids. Note: scale bar distances are shown in each panel. N: cell nucleus; SG: secretory granule; and CF: collagen fibers

The ability of cell aggregates to further develop into organoids was examined using a subrenal capsule implantation assay (Fig. 7). Cell aggregates (*n* = 200–300) were collected from 14-day cultures and surgically implanted. As a negative control, untreated 14-day BM-MSCs were mixed with Matrigel and implanted using the same surgical procedures. After 14 days of implantation, the region where the cell aggregates had been placed developed a white protuberance in situ*.* This mass continued to develop and increase in size through day 30 (Fig. 7A). In contrast, mice receiving the Matrigel-encased BM-MSCs did not develop any obvious tissue-like mass.

Histological analysis of the 14-day post-implantation specimens revealed that the mass, a combination of cells and ECM, resembled the cell aggregates formed in vitro (see Fig. 4A) and did not form any SG-like structures (Fig. 7B). In contrast, the day 30 implants contained a number of secretory acini-like units. In the photomicrographs, an outlined area delimits a cluster of cells surrounding a central lumen and includes nuclei located peripherally against the cell membrane (Fig. 7B, day 30 implants). In addition, a number of blood vessels between the acini-like units were observed in H&E-stained sections, presumably due to de novo angiogenesis (Fig. 7B). Moreover, frozen sections of the implants stained negatively with Oil Red O, confirming that fat/oil droplets, indicative of adipose tissue, were not present (data not shown). PAS staining identified the presence of numerous (~ 20%) positively stained cells in both day 14 and day 30 implants (Fig. 7B). Trichrome staining (in blue) revealed the presence of collagen fiber bundles in day 14 implants and by day 30 identified a more organized collagenous matrix surrounding the secretory units (an area delimited by the box), suggesting that a basement membrane was developing. Immunofluorescence staining showed strong expression of SG-specific markers including amylase, Krt14, and the tight junction protein Cldn10; notably, Aqp5^+^ acinar progenitor cells were only found in the 30-day implants (Fig. 7C). TEM images further confirmed that the cells within the implants contained numerous secretory granules and some cells were surrounded by collagen fibers (Fig. 7D).

## Discussion

Stem cell-based repair or regeneration of the SG is a challenging task because the resident stem cell population is limited and not well characterized [16, 17]. Previously, we reported that tissue-specific decellularized ECMs direct multipotent BM-MSC differentiation to the same lineage as that of the ECM [24]. To demonstrate “proof of concept” in the current study, we showed that differentiation of BM-MSCs can be guided to the SG epithelial cell lineage by incubation with SMG-ECM (but not BM-ECM) and form cell aggregates that express SG biomarkers, generate intracellular secretory granules in vitro, and develop SG-like organoids in vivo.

Decellularization of SMG tissue is an important step in preparing ECM homogenates since cellular constituents must be removed, while the critical components of the ECM/matrix are retained [34]. The decellularization method we employed was modified from previous reports [26, 27] and successfully removed most of the SMG cell components and maintained the integrity of the protein matrix.

The proteomic analysis performed in this study employed a comprehensive gene set enrichment program (Enrichr) to classify proteins identified in SMG [35], before and after decellularization, based on tissue ontology. Our analysis indicated that a large number of SG components and a small fraction of ECM constituents were removed by the decellularization process, while most major ECM structural and basement membrane proteins remained in the decellularized SMG tissue. These results are consistent with previous reports indicating that the remaining proteins are important components of native SMG tissue [36, 37] and play key roles in the development and maintenance of SG function [38, 39].

Interestingly, the decellularized SMG tissue contained 171 unique protein components, only 9% of which were ECM constituents. This result may be explained by (1) differences in protein extraction procedures between whole SMG tissue and decellularized SMG tissue (the former was prepared by homogenization only, while the latter was first decellularized and then homogenized); (2) removal of numerous SG proteins may have reduced protein assignment ambiguity from peptide fragments associated with ECM proteins; and (3) donor variation in SMG tissue samples. Nevertheless, SLRPs (e.g., decorin, biglycan, and asporin), which were dramatically enriched in decellularized SMG tissue, have been found to play important roles in cell–matrix interactions and modulating growth factor activity [40]. However, a role for SLRP family members in regulating BM-MSC trans-differentiation to the epithelial cell lineage requires further investigation.

To specifically assess the effect of decellularized SMG-ECM on BM-MSC trans-differentiation, the expression of a series of specific SG epithelial cell lineage markers was examined. The results showed that *Muc10*, an early marker of mucous acinar cells [41–43], was detected in the aggregate cells during the first 7 days of culture, while more mature SG epithelial cell markers, including *Krt14* (a marker for intercalated ductal cells) [44, 45], *Mist1* (a transcription factor found in serous exocrine cells) [46], and the tight junction proteins *Cldn3* and *Cldn10* (expressed by SG acinar cells) [47], were expressed (in addition to *Muc10*) during the second 7 days of culture. The presence of differentiated cells in the aggregates was further confirmed by PAS staining, which indicated the presence of secretory glycoproteins or mucin-like substances. In addition, the expression of SG-specific lineage markers (i.e., amylase, Aqp5, Mist1, Krt14, Cldn3, and Cldn10) at the protein level was demonstrated by immunofluorescence staining. More importantly, our observation of intracellular secretory granule-like structures and peripherally located nuclei in aggregate cells suggested that the differentiated cells were involved in exocrine functions [48–51]. In addition, there were tight junctions between adjacent cells. However, none of these ultrastructural characteristics were detected in untreated BM-MSCs, SMG-ECM-treated monolayer cells, or BM-ECM-treated cells. Taken together, these results indicate that incubation of BM-MSCs with SMG-ECM leads to the formation of cell aggregates containing cells that have trans-differentiated to the SG epithelial cell lineage.

While a small proportion of BM-MSCs pretreated with the SMG-ECM homogenate formed cell aggregates, the vast majority formed a monolayer on the plastic tissue culture surface and failed to differentiate into SG epithelial cell progenitors. To determine the phenotype of cells present in the aggregates, we characterized the cell population for expression of precursor lineage markers: CD90 (MSCs), CD105 (vascular endothelial cells), and CD133 (epithelial cell precursors) [22]. We found that CD133 expression was highest (~ 60%) and CD90 lowest (~ 45%) in aggregate cells from day 14 cultures, compared to the initial population of BM-MSCs (P1), untreated BM-MSCs, and SMG-ECM-treated monolayer BM-MSCs. To further determine whether the subpopulation of CD133+ BM-MSCs is more effective at inducing cell aggregation and SG differentiation, we cultured purified CD133+ and CD133− cells. Indeed, CD133+ cells formed substantially more cell aggregates than cultures initially seeded with SMG-ECM-treated CD133− cells. Further differences were revealed by PAS-positive staining and the presence of secretory granules in the cytoplasm. Interestingly, it has been demonstrated that CD133 (also known as prominin-1) is a SG stem cell marker [11, 52] which further supports our observation that these cells respond to incubation with SMG-ECM by trans-differentiation into SG progenitors and suggests a potential translational approach for achieving SG regeneration and repair.

To demonstrate that cell aggregates have the ability to undergo SG organogenesis, we implanted them into the renal capsule of immunocompromised mice for 14 and 30 days. This in vivo animal model has been used to study early development of secretory organoids [53, 54]. At day 14 post-implantation, some cells were already expressing SG-specific markers. Remarkably, by day 30 post-implantation, cells were found to form secretory structures consisting of ductal and acinar-like cells with tight junction proteins. Immunofluorescence staining demonstrated strong staining for Krt14, Aqp5 [55], Cldn10, and amylase, suggesting that the SG organoid was functional. In addition, many newly formed blood vessels were observed around and penetrating the developing tissue. Unfortunately, we were unable to detect the presence of any mucosubstances (i.e., Alcian blue positive) in the lumen of secretory units, probably lost during paraffin embedding and sectioning. These strikingly positive results demonstrate that BM-MSCs trans-differentiated into SG progenitor cells in vitro, which can further differentiate/develop into SG organoids in vivo.

Accumulating data indicate that autologous MSCs are preferable to allogeneic MSCs for stem cell-based tissue regeneration due to biosafety concerns and increasing evidence suggesting that allogeneic MSCs may not be immune privileged [56, 57]. Previously, our laboratory has shown that BM-MSCs from older patients, with diminished quality and quantity, can be rejuvenated by culture on young ECM (microenvironment) [32, 33]. In the present study, we propose a new paradigm for repairing damaged SGs using autologous MSCs. By combining our stem cell rejuvenation and tissue-specific ECM induction technologies, autologous BM-MSCs from elderly patients could be used to produce sufficient quantities of SG epithelial cell progenitors for use in repairing their damaged SGs.

Several questions, emanating from the results of this study, remain to be addressed in future studies. For example, what are the key effective components in SMG-ECM that promote trans-differentiation of BM-MSCs to the SG epithelial cell lineage and ultimately generate SG organoids? Moreover, how efficacious are cell aggregates in repairing SG damage? At present, we are establishing a rodent model of radiation-induced SG injury in our laboratory to test the efficacy of the cell aggregates [11], but even if efficacious it will require additional human studies to translate the results to the clinic.

## Conclusions

In conclusion, the present study proposes the use of decellularized SMG-ECM as an innovative approach for promoting the trans-differentiation of BM-MSCs to the SG epithelial cell lineage. The resulting cell aggregates express typical SG epithelial cell characteristics in vitro and create SG-like organoids when implanted in vivo. The results suggest the feasibility of using autologous BM-MSCs as an abundant source of stem cells for treatment of SG dysfunction.

## Data Availability

Data supporting the results described in this study can be obtained by request from the corresponding authors.

## Abbreviations

BM-MSCs: Bone marrow-derived mesenchymal stem cells
BM-ECM: Bone marrow stromal cell-derived extracellular matrix
Cldn3: Claudin 3
Cldn10: Claudin 10
ECM: Extracellular matrix
GAPDH: Glyceraldehyde-3-phosphate dehydrogenase
Krt14: Cytokeratin 14
Mist1: Muscle, intestine, stomach expression-1
MSCs: Mesenchymal stem cells
Muc10: Mucin 10
SG: Salivary gland
SG-ECM: Salivary gland extracellular matrix
SMG: Submandibular gland
SMG-ECM: Submandibular gland extracellular matrix
